# Whither RDS? An investigation of Respondent Driven Sampling as a method of recruiting mainstream marijuana users

**DOI:** 10.1186/1477-7517-7-15

**Published:** 2010-07-09

**Authors:** Andrew D Hathaway, Elaine Hyshka, Patricia G Erickson, Mark Asbridge, Serge Brochu, Marie-Marthe Cousineau, Cameron Duff, David Marsh

**Affiliations:** 1 Department of Sociology and Anthropology, University of Guelph, Guelph, Ontario, Canada, N1G 2W1; 2 Community-University Partnership for the Study of Children, Youth and Families, University of Alberta, Edmonton, Alberta, Canada; 3 Department of Social, Prevention and Health Policy Research, Centre for Addiction and Mental Health, Toronto, Ontario, Canada; 4 Department of Community Health and Epidemiology, Dalhousie University, Halifax, Nova Scotia, Canada; 5 Centre International de Criminologie Comparee (CICC), University of Montreal, Montreal, Quebec, Canada; 6 Social Sciences and Health Research Unit, School of Psychology, Psychiatry and Psychological Medicine, Monash University, Melbourne, Victoria, Australia; 7 Addiction Medicine, Vancouver Coastal Health, Vancouver, British Columbia, Canada

## Abstract

**Background:**

An important challenge in conducting social research of specific relevance to harm reduction programs is locating hidden populations of consumers of substances like cannabis who typically report few adverse or unwanted consequences of their use. Much of the deviant, pathologized perception of drug users is historically derived from, and empirically supported, by a research emphasis on gaining ready access to users in drug treatment or in prison populations with higher incidence of problems of dependence and misuse. Because they are less visible, responsible recreational users of illicit drugs have been more difficult to study.

**Methods:**

This article investigates Respondent Driven Sampling (RDS) as a method of recruiting experienced marijuana users representative of users in the general population. Based on sampling conducted in a multi-city study (Halifax, Montreal, Toronto, and Vancouver), and compared to samples gathered using other research methods, we assess the strengths and weaknesses of RDS recruitment as a means of gaining access to illicit substance users who experience few harmful consequences of their use. Demographic characteristics of the sample in Toronto are compared with those of users in a recent household survey and a pilot study of Toronto where the latter utilized nonrandom self-selection of respondents.

**Results:**

A modified approach to RDS was necessary to attain the target sample size in all four cities (i.e., 40 'users' from each site). The final sample in Toronto was largely similar, however, to marijuana users in a random household survey that was carried out in the same city. Whereas well-educated, married, whites and females in the survey were all somewhat overrepresented, the two samples, overall, were more alike than different with respect to economic status and employment. Furthermore, comparison with a self-selected sample suggests that (even modified) RDS recruitment is a cost-effective way of gathering respondents who are more representative of users in the general population than nonrandom methods of recruitment ordinarily produce.

**Conclusions:**

Research on marijuana use, and other forms of drug use hidden in the general population of adults, is important for informing and extending harm reduction beyond its current emphasis on 'at-risk' populations. Expanding harm reduction in a normalizing context, through innovative research on users often overlooked, further challenges assumptions about reducing harm through prohibition of drug use and urges consideration of alternative policies such as decriminalization and legal regulation.

## Background

The widespread use of cannabis (Cannabis sativa/indica and related species also widely known as 'marijuana') in many western countries far exceeds the prevalence of other illegal drugs [1]. Despite mainstream diffusion of the practice, there are few qualitative studies of 'ordinary,' functioning, socially-integrated users who hold jobs, raise families and exhibit stable lifestyles [2-6]. Compared to other studies of more easily located youth and young adults in university or high school [7,8], qualitative studies of marijuana use among adults are based primarily on samples that are narrow, self-selected, already publicly identified, and attracted by the offer of a payment to take part [9]. The illegality of marijuana use is in itself a disincentive. Those most likely to participate presumably have less to lose by the disclosure, may need the money more than others, or develop trust in a specific interviewer. Participants may also have more formal education, and thereby place more value on research. However, samples vary widely by the method of recruitment and are rarely generalizable to 'marijuana users' overall. And whereas studies based on population surveys have produced samples more closely representative of mainstream populations, such methods are expensive and not typically conducive to unstructured interviewing and other forms of qualitative research [10].

Although convenience samples have provided needed insights into the 'deviant' subculture of marijuana use, we set out to generate a sample of respondents hidden in the general population of adults. Our research questions and hypotheses are guided by the proposition that cannabis has undergone a *normalizing *process [11-13] as indicated by high use rates, easy access, social tolerance, and accommodation of the practice by nonusers. Thus, we speculated, if the target population's experience of stigma is substantially reduced, users are accordingly more open to the prospect of disclosure of this status for the purpose of research. Moreover, in our study, consistent with this thesis, 'normal' users are an understudied group well worth pursuing to expand the knowledge base on marijuana use. Such research is vital to inform the debate about replacing or modifying prohibition with a harm reduction policy. Criminal sanctions are a costly and particularly harmful option when applied to productive, otherwise law-abiding individuals [9]. After much deliberation, ethical review, and piloting of our recruitment method, we settled on an adaptation of Respondent Driving Sampling, a method previously employed in other studies of drug users [14-16] but never for recruiting 'mainstream' marijuana users.

In this paper, we review the literature and our own experience with RDS and demographic profiles of participants recruited in four cities across Canada. To critically assess the representativeness attained through our adapted RDS approach, we then compare the sample that was gathered in Toronto with those from two prior studies of marijuana users that also were recruited in the city of Toronto. The first of these was randomly conducted via household survey of respondents in the general population [17]. The second was a pilot project that relied on recruitment of respondents from a local free newspaper, which resulted in a sample that was biased with respect to more use and problematic use, and other characteristics such as lower income and employment [18,19]. This three-way comparison of sample demographics, derived with different methods by studies in the same location [cf. [20,21]], sheds light on strengths and weaknesses of RDS recruitment as a method of researching mainstream marijuana users.

## Methods

### Respondent Driven Sampling

Hidden populations are characterized by certain features that make their members difficult to study and make estimates about their demographic composition; these may include the lack of sampling frame, small size of population, the experience or anticipation of stigma among members, and reluctance to share information with outsiders [22]. These characteristics often stem from the illegality of the activity and likelihood of social disapproval if discovered. Probability sampling in hidden populations is impractical and technically impossible, precluding the gold standard for collecting unbiased quantitative data [23,24]. Past studies have relied upon nonrandom sampling methods like convenience sampling and snowball/chain-referral that can yield large samples yet offer no assurance of the representativeness of findings. Targeted (in time/space or venue-based) sampling are variations often used when hidden populations are concentrated in a given geographic region [24].

Ethnographic mapping of the target population may be combined with interviews with local key informants to further guide the process of recruitment. Chain-referral sampling is more suitable when members of the hidden population are connected via social networks as opposed to geographical locations. Despite these adaptations, nonrandom methods of selection are criticized as biased insofar as certain segments of the population are inaccessible for sampling [25]. Other common forms of bias are demographic sameness, volunteerism, masking (peers protected by participants refusing to refer them), and differential recruitment--one peer group overrepresented or underrepresentation of those who are less socially connected [24].

To address these types of biases, Douglas Heckathorn [25] developed respondent driven sampling as a chain-referral method that relies on 'contact patterns' of routine interaction among the social networks in a hidden population [14]. If the pattern of referral has been closely tracked and modeled, "it is possible to derive statistically valid indicators and quantitatively determine their precision" [24]. In order for inferences about a hidden population to be asymptotically unbiased, RDS relies upon particular procedures and strict adherence to the sampling criteria [26].

Members of the population are first purposively selected. These 'seeds' are interviewed and then requested to recruit a set number of their peers via a numbered coupon system. The interviewer gathers information from respondents about the size of their respective networks. The number of coupons each participant is given reflects a recruitment quota that prevents over-recruitment by more socially connected seeds of peers within their network. Recipients of coupons who contact the researcher are screened for eligibility and interviewed. This process repeats itself through a series of recruitment waves and the sample geometrically expands. A series of financial incentives is employed for participation and additional recruitment to minimize attrition in the sample. If successful, after several waves a point of equilibrium is reached in which the sample characteristics approximate parameters of the population.

The coupon system is important to enable precise tracking of who recruited whom and their number of social contacts. A mathematical model of the entire recruitment process is used to weight the sample and compensate for non-random patterns of recruitment [27]. Unbiased population estimates are thereby generated and measured for precision. Moreover, RDS allows researchers to assess the "measures of affiliation, or the degree of connection between members of different groups, [which] can be used to conduct analyses of the social structure of the hidden population under study" [16].

First employed by Heckathorn to study HIV risk behaviors among injection drug users (IDUs) in the United States [25], RDS has since been used in many types of studies of at-risk populations that are difficult to reach--for example, IDUs, sex workers, men who have sex with men, and other groups at elevated risk of HIV among other infectious diseases [14,16,23,28-31]. Other applications include Heckathorn and Jefferi's research on jazz musicians [22,32], suggesting these procedures can be fruitfully adapted for purposes of study of a wide variety of hidden populations with more or less experience of stigma.

The most significant advantage of RDS reported is elimination of known biases, thus yielding (with large samples) statistics fit for inference to hidden populations [24]. RDS allows for an analysis of social structures based on access to some segments of the hidden population that are inaccessible via other methods [16,22,24]; and researchers can vary the pace of recruitment and control for underrepresentation of some segments [32]. But there are criticisms of a method of recruitment that is so reliant on providing cash incentives.

For example, Scott describes how RDS resulted in an underground economy involving sale of coupons among injection drug users in a Chicago study [33]. He reported instances of violence, coercion, false reports of drug use among 'eligible' respondents, and suspected sero-mixing of IDUs with HIV and HIV-negative users. Scott points out that RDS necessitates the breach of confidentiality, since subjects cannot participate in the recruitment process without at least one peer within their social network knowing. Another disadvantage is the need for self-reporting of network size by members of the hidden population. The large potential for error requires that researchers remain vigilant and exercise great caution to maximize the accuracy of these important estimates [15].

While Scott's critique [33-38]--and Heckathorn's statistical assumptions [20,26,39-42]--have invited vigorous, continuing debate, the literature on RDS is generally supportive of its use with hidden populations like our own. After considering the options available for sampling marijuana users from the general population, either from a survey or more traditional snowball sampling, we selected RDS as our recruitment method. It promised a novel, cost-effective approach of sampling an understudied population of drug users and producing a more representative, socially integrated sample of adults than the other options we considered.

### The four-city study: A modified approach

Apart from just one study about cannabis dependence that recruited through the use of posters [43], to our knowledge RDS has never been adapted for research on marijuana users. Because our protocol and budget called for only 40 cannabis users per site (and 10 tobacco users in each city for comparison), the RDS requirements for statistical analysis were not met by the final sample sizes in this study [24-27]. We nonetheless aspired to follow sampling conventions of the RDS recruitment method. Rather than achieving strict representativeness in terms of generating data that are suitable for inference, we settled on the conduct of a chain-referral method that is innovative and potentially improves on other methods of recruiting mainstream marijuana users.

Compared to other smaller populations of drug users, marijuana users are numerous but less likely to be linked to geographical locations; this makes it hard to find them 'in the field' [1,44]. Therefore each of the four sites began with marijuana users located in the local social networks of team members. We purposively sought socially well-integrated users, defined as adults between 20 and 49 years of age, employed or in school, and in stable housing for the past six months. The threshold for 'regular' use was defined as twice a month on average over the past five years. Whereas more diversity in seed selection would have been preferred in our recruitment process, the breadth of initial contacts was encouraging and seemed to justify persisting with the method.

Initial seed-participants completed a brief survey (10-20 minutes) with one of the team members, and about an hour-long semi-structured interview. Following the interview all seeds were offered printed cards with contact information and a description of the study to pass on to anyone they knew who met the study criteria. All participants were paid $20 for their time, regardless of their willingness to pass out referral cards to others. For each successful referral (up to three peers) we offered an entry in a draw, with winners notified by email, for a gift certificate worth $500. Given the demographics of our sample, we assumed that the chance to win a shopping spree at a local mall was more enticing than the offer of cash payment that is typically extended (around $10/referral) in RDS recruitment of more marginalized respondents.

**Figure 1:**
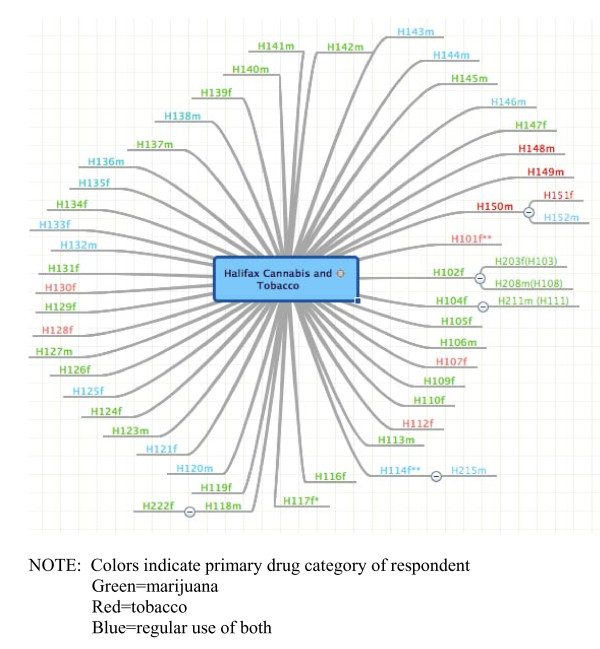
**Recruitment diagram for Halifax**.

**Figure 2:**
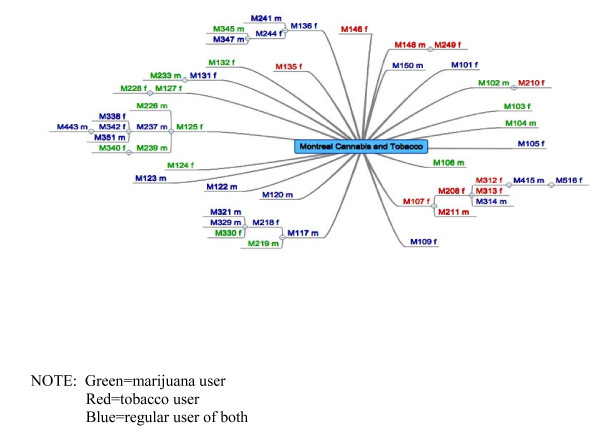
**Recruitment diagram for Montreal**.

**Figure 3:**
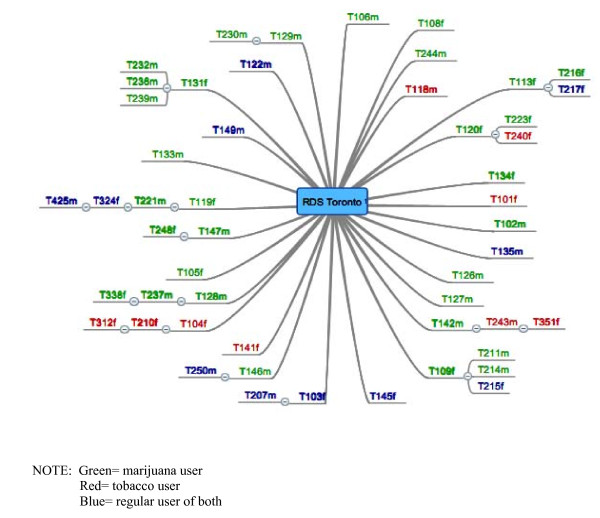
**Recruitment diagram for Toronto**.

Gaining ethical approval for a common methodology in all 4 cities proved to be a challenge. The Research Ethics Board at one site did not approve our incremental method of providing more incentive for recruitment, viewing it as coercive. Accordingly, in that city, participants were given one entry in the draw regardless of the outcome of their peer recruitment efforts. A concern in another site related to having the title of the project ("drug normalization and stigma study" without specifying the drugs) on the card to be given out. The REB expressed concern that mentioning these terms would be a risk to participants if discovered by the 'wrong' person. Similarly, the REB at one site had objected to the idea of using potentially identifying email accounts to notify winners of the draw. Thus, to be consistent, all four sites implemented the option for participants to create an anonymous email account and later notify us of the designated address. The wide variety of REB responses we encountered suggests a need for dialogue with REBs regarding complexities and challenges of RDS recruitment [44]. Ultimately, the participants and their referrals were identified by serial numbers printed on recruitment cards and tracked by the researchers with a digital mapping tool that provides a visual record [see Figure 1, Figure 2, Figure 3]. The chain-referral process was extended and repeated as each new participant was asked to refer up to three peers to participate, and so on.

All sites began recruitment in July or August 2008 after receiving local REB approval, and piloting the survey and interview schedules. Despite success locating local seeds who indicated use of cannabis within their social networks, recruitment progressed slowly at all sites. After several months less than ten respondents were recruited in all cities, other than Toronto which fared better with 18. Accordingly, the protocol was altered to allow for other recruitment strategies to boost the sample sizes. The addition of new research assistants in Toronto and Halifax provided later seeds, and we removed the three-peer quota to permit participants by word-of-mouth referral. Based on feedback from participants that this process was more arduous, time consuming, or imposing than initially expected, we also sent reminders via e-mail to respondents who agreed to help us with recruitment; but this was not particularly successful. Since most of the participants worked full-time outside the home, the hours available for contact were also often limited which made scheduling the interviews more difficult to manage.

In addition to exhausting all the contacts in their networks, the different sites relied on other forms of advertisement such as developing a website (Montreal) and strategically placed posters (Vancouver and Halifax). In sum, despite our efforts and commitment to the method, the RDS approach required substantial adaptation that led to inconsistency in our recruitment strategies. We also note large differences between the sites regarding successful propagation of the first-wave seeds selected. In Halifax, just seven of the final sample gathered (n = 49)--including both the cannabis and tobacco users--were brought to the study by way of chain-referral. Likewise, in Vancouver only twelve were peer-recruited; the recruitment diagram (not included) resembles Figure 1. By contrast, in Montreal less than half the sample (n = 22 of 50) is comprised of their initial seeds. Toronto's map is similar (with 23 of 51 obtained by chain-referral), but few 'chains' in either city generated more than one additional referral.

The modified approach to RDS we implemented violates assumptions and statistical requirements on which the method's claims to representativeness are based. Apart from higher budgets to facilitate large samples, the success of RDS is naturally contingent on the facilitation of successful peer recruitment. Put otherwise, ideally, more developed chains in each site would have yielded samples that are less heavily comprised of initial seeds recruited by the research team directly, which would remove the need for other forms of advertisement. Notwithstanding these shortcomings, and emergent adaptations that were needed to complete the study, the final sample characteristics are especially instructive when looked at in comparison to samples that were gathered in studies implementing other methods of recruitment.

### Demographic profile of respondents in four cities

Table 1 presents the demographic characteristics of marijuana users in the study we recruited through use of a modified RDS (MRDS) approach. The age range (20 to 49 yrs.) and mean (29-31) are consistent between sites, with slightly fewer female respondents in most cities and significant group differences in sexual orientation. Over a third in Vancouver identified as being bisexual or homosexual (37%), compared to the much lower rates that varied widely elsewhere--Montreal (2.5%), Toronto (9.5%), and Halifax (17%). Most were born in Canada, with little variation between the sites in ethnic representation. Respondents in Vancouver more often reported their ethnicity as 'other,' and were more likely to have moved there from another province. Most notably, the same number of participants living in Vancouver listed their birth province as Ontario as those from British Columbia (the western province where the city of Vancouver is located).

**Table 1:** Demographic characteristics of respondents in four cities

	Vancouver (N = 41)	Toronto (N = 42)	Montreal (N = 40)	Halifax (N = 42)
mean age	31.6 yrs. (SD 8.3)	30.6 yrs. (SD 7.2)	28.9 yrs. (SD 6.1)	30.7 yrs. (SD 8.7)
% male	63	57	55	48
% married or common law	42	48	48	40
% born in Canada	93	83	92	98
% white/European background	88	86	98	79
% completed univ. or college	58	81	72	48
% working full-time	42	60	68	48
% working part-time	20	10	12	12
% >$50,000 household income	34	50	48	24
% renting house or apartment	83	71	62	74

There are significant group differences in education levels--more with university and postgraduate degrees in Toronto (64%) as compared to other cities (which range from 38 to 58%). One in three in Halifax (36%) reported high school only versus only 7% in the Toronto group. More were fully employed in Montreal and Toronto compared to the other two cities, and differences in annual household income were substantial. Half as many in Toronto (24%) reported less than $35,000, relative to Halifax (50%) with more moderate group differences between Montreal (40%) and Vancouver (44%).

Nearly everyone in all sites considered their housing "stable" (from 88% in Montreal to 100% in Vancouver), with most respondents renting a house or an apartment. Fewer in Vancouver (15%) owned their own home as compared to Montreal (32%), Halifax and Toronto (both 25%). The proportion of participants who were married or living with a partner was similar across the research sites (40-48%). Yet there were twice as many 'singles' in the study in Toronto than Montreal (45 vs. 22.5%)--as compared to one in three in Halifax (31%) and Vancouver (32%).

Ultimately, we succeeded in recruiting at least 40 marijuana users in each of the four cities. Despite some variation in demographic characteristics, the sampling criteria for age range and employment and stable living conditions were achieved. Thus we have some confidence that we have tapped into the less visible majority of marijuana users 'hiding' in the mainstream population of adults. To assess the representativeness of the MRDS with respect to users in the general population, we restrict our focus now to the Toronto sample compared to other samples that were gathered in Toronto in two previous studies using different research methods. Specifically, the demographic profile of respondents is compared with that of users in a random household survey and another study in Toronto that recruited through nonrandom self-selection of respondents.

### Comparing demographic characteristics in three studies

To facilitate comparison across the different studies, we selected the most frequent, current marijuana users. From each of the three samples we included only those who used cannabis more often than once a week on average in the 30 days before the interview or survey. This reduced the sample size of the MRDS from 42 to 36 respondents in Toronto, one-third of whom used daily during the past month. The same criterion of more than once a week in the past month resulted in a sample size of 51 respondents (half used daily) selected from a random household survey of Toronto [17]. That study's method of recruitment (and that of the third study) is described in brief before comparing demographics and discussing the potential implications for research.

The household survey of Toronto we refer to was conducted to measure public attitudes regarding marijuana use and opinions on drug policy reform. In October-November 2004, interviewers from a university-based survey research center telephoned randomly generated numbers for households (and cell phone subscribers) in Metropolitan Toronto (416 exchange). They asked to speak to the person 18 or older whose birthday was nearest the day of the call. In addition to the standard demographic information included in Ontario's provincial drug use survey [45], other items asked about the use of marijuana (e.g., Ever used? If so, how many times? How often in the past year, and over the past month?).

Of 5000 numbers dialed, 1440 (28.8%) households were successfully contacted and definitively yielded an eligible respondent. A total of 1081 fully completed the survey, for an overall response rate of 75%. The demographic profile is generally consistent with that of the Toronto sub-population surveyed in the Ontario Drug Monitor of 2004-05 [45] with university educated and female respondents being somewhat overrepresented in the survey. One-half of those surveyed (527) used marijuana at least once, with 80% of this group (420) reporting past-year use and 23% reporting use in the past month (122). Fifty-one respondents in the latter group reported using marijuana more than once a week on average (half of whom used daily) in the last 30 days.

To augment the analysis of demographic profiles, in contrast to MRDS and random phone recruitment, a study from Toronto with nonrandomized recruitment was compared on sample demographics with these others. Respondents were recruited through a local free newspaper advertisement seeking ''experienced'' cannabis users, 18 years or older, having used 25 or more times throughout their lives [12,18,19]. Approximately 200 persons left telephone messages expressing interest in the study, nearly three quarters of whom were successfully contacted and willing to participate in a private interview. One hundred and four kept their designated appointments to conduct an in-depth interview at a downtown research office between October 2000 and April 2001. Despite its nonrandom design limitations, respondent self-selection proved advantageous in this study as a cost-effective method of attracting more committed, long-term, frequent users to take part. An honorarium of $25 was offered to compensate participants for their time and contribution to the study.

While many said they came due to their interest in the research, the cash incentive influenced the demographic profile and income distribution of the sample. For example, 82% earned less than $2000 a month (net income) in the previous tax year, and 36% earned less than half that modest income. Forty-one percent worked full-time (35 or more hours per week), while 12% were full-time students, and one in four (24%) received some form of public assistance. Thus the sample is acknowledged to be over-representing users with higher frequency of use and lower income. Indeed (in contrast to the aims of the MRDS to target fewer marginalized, more "integrated" users), this sample is both skewed in terms of economic status and, compared to random samples drawn from population surveys [10,46], used more cannabis more often than those in other studies. Using the criteria of frequency adopted of use of more than once a week over the past month, 75 respondents (two-thirds of whom used daily) were selected from the sample in order to compare them with the other study groups.

## Results and Discussion

Table 2 presents selected demographic data for each of the three samples of marijuana users recruited in the city of Toronto. Three years younger on average, the MRDS sample included twice as many female marijuana users (42% vs. 20%) as the household survey in the city of Toronto that was derived by Random Digit Dialing (RDD). The oversampling of females is a strategy adopted in a wide variety of studies of drug users. Threats to representativeness are arguably outweighed by the benefit of gaining a better understanding of gender differences in patterns and experiences of use.

**Table 2:** Demographic characteristics of respondents in three studies of Toronto marijuana users

	MRDS respondents (N = 36)	RDD respondents (N = 51)	Self-selected respondents (N = 75)
mean age	31.3 yrs. (SD 7.4)	34.4 yrs. (SD 12.6)	32.4 yrs. (SD 8.9)
% male	58	80	63
% married or common law	44	31	16
% born in Canada	83	76	76
% white/European background	89	74	NA
% completed university or college	70	39	NA
% working full-time	56	76	41
% working part-time	11	10	27
% Currently in school	33	16	17
% >$50,000 household income	50*	54**	NA***

*average annual household income

**2003 household income before taxes

***2000-01 personal monthly take-home income

Most notably, consistent with the MRDS objective of recruiting socially well-integrated users, the economic status of respondents is reflective of the higher incomes found when users are recruited from the general population of the city of Toronto. Much like the household survey found, the annual household income of roughly half the sample was over $50,000. This compares (though imprecisely) with the 'self-selected' sample in which only 9% reported (personal) take-home income exceeding $2,000 a month. While more than half (56%) worked full-time in the MRDS study, the less employed (working part time or in school) are, nonetheless, still over-represented as compared to users in the random household survey.

Respondents born in Canada are also over-represented, as are married persons, those of European background, and graduates of university or college. Considering our emphasis on 'mainstream' types of users, our modified approach to RDS was a success. That is, it proved successful in Toronto for producing a small sample that is similar on certain demographics to one achieved through random digit dialing. At the same time, 'representativeness' has not been demonstrated conclusively by any means, in terms of the 'gold standard' that a randomized design presumably reflects. Underestimating stigma or diversity, or other implications of the type of bias this suggests, has further implications for the development of theory on normalizing processes as well. Clearly, more and better research in the mainstream population is needed for development of harm reduction programs based on actual perceptions and experiences of users hidden in the general population of adults.

A modified approach to RDS was necessary to attain the target sample size in all four cities. The coupon strategy was relaxed, for example, to allow for posted ads and word-of-mouth referral. The additional incentive for referral of one's peers (the $500 'draw') was not especially successful as a means to overcome whatever barriers to taking part there may have been, including lack of time or interest, or the need to be discreet. Moreover, the logistics of requiring use of coupons, and lack of a financial need within this population, appeared to greatly hinder the success of this approach. It remains a quandary for future research to consider whether larger sums, or more immediate cash payments, or other ways to stimulate more interest in a draw, could (should?) be used to motivate more active peer recruitment.

The protocol was also met with varying resistance by the Research Ethics Boards at the respective sites. Consistency was difficult to maintain throughout this process which posed another challenge for the study. Ethical concerns regarding RDS recruitment are important issues to be dealt with case-by-case, to foster uniformity where possible and practical, and to build consensus with respect to research standards and practice across different academic institutions. Unlike other studies of more marginalized drug users [38], there is no evidence respondents had been pressuring their peers or in any way coerced them to take part. Rather, on the contrary, the study generated insufficient interest in peer networks to sustain the research team's adherence to strict RDS procedures.

What level of incentive is required to motivate more interest in the target population for such research? Whereas most marijuana users appear to meet the definition of a hidden population, with routine interactions in their various networks, RDS is difficult with 'wealthier' more mainstream segments of drug using populations. Perseverance ultimately paid off in this study, after the adoption of a modified approach. Future studies in this vein on mainstream substance users should explore developing more appealing incentives to overcome disinterest or resistance to research. These demands are countered by the risk of being judged overly 'coercive' by the Research Ethics Board. While dilemmas of this type are common in most research protocols involving human subjects, resolving them is critical to the success of studies using RDS recruitment and other innovative methods to access hidden segments of drug using populations for purposes of harm reduction oriented research.

## Conclusions

Comparing socio-demographic characteristics of three samples of marijuana users in the city of Toronto, we found that the MRDS-derived one is a closer reflection of respondents in a random household survey than a simple 'self-selected' sample. In terms of representativeness, for qualitative research, this method of recruitment may thus be a cost-effective alternative to population surveys that improves on advertising and respondent self-selection. More in-depth exploration of the normalization thesis will require more research that is able to tap into socially well-integrated networks of drug users. RDS in this respect is largely advantageous but also has been shown to have important limitations that necessitated changes to resolve them in our study, and attain the target sample sizes in all sites.

Much of the evidence to date on normalization has been based on narrow samples of respondents who are students--young adults and adolescents in university or high school [13]. Investigating widespread societal diffusion of a normalizing process around the use of drugs requires a broader age range to provide a fuller test. Sensible, controlled use of illicit drugs may also include a wide variety of substance use that does not correspond with common understandings of drug use(rs). Despite the many challenges encountered in this study, respondent driven sampling, of one form or another, is a cost-effective way of gathering respondents who are otherwise invisible consumers of these drugs. Lessons learned from RDS with marijuana users may extend to other substance users in the mainstream, and other forms of law breaking or risk taking behavior. In any case, appropriate incentives and concern for coercion of respondents must be weighed when seeking access to potential subjects in the general population.

Demonstrating that 'normal' substance users represent large segments of both mainstream and 'drug-using' populations erodes the justification for prohibition and contributes to the evidence base for a public health approach [47]. The experiences of users who appear to have few problems of the type attributed to regular drug use are equally important to inform our understanding of substance use and misuse in a harm reduction framework. The challenges of research in this normalizing context [48], and changing demographic profile of illicit substance use, requires more innovative and adaptive study methods to generate more samples representative of users in the mainstream general population of adults.

## Competing interests

The authors declare that they have no competing interests.

## Authors' contributions

ADH conducted the comparative analysis and drafted the manuscript, with contributions to the writing from EH and PGE. All authors participated in the design of the study and collection of the data at the respective research sites. All authors read and approved the final manuscript.
